# Metal contamination in harbours impacts life-history traits and metallothionein levels in snails

**DOI:** 10.1371/journal.pone.0180157

**Published:** 2017-07-03

**Authors:** Maria Alexandra Bighiu, Elena Gorokhova, Bethanie Carney Almroth, Ann-Kristin Eriksson Wiklund

**Affiliations:** 1 Department of Environmental Science and Analytical Chemistry, Stockholm University, Stockholm, Sweden; 2 Department of Biological and Environmental Sciences, University of Gothenburg, Gothenburg, Sweden; CINVESTAV-IPN, MEXICO

## Abstract

Harbours with limited water exchange are hotspots of contaminant accumulation. Antifouling paints (AF) contribute to this accumulation by leaching biocides that may affect non-target species. In several leisure boat harbours and reference areas in the Baltic Sea, chronic exposure effects were evaluated using caging experiments with the snail *Theodoxus fluviatilis*. We analysed variations in ecologically relevant endpoints (mortality, growth and reproduction) in concert with variation in metallothionein-like proteins (MTLP) levels. The latter is a biomarker of exposure to metals, such as copper (Cu) and zinc (Zn), which are used in AF paints as active ingredient and stabilizer, respectively. In addition, environmental samples (water, sediment) were analysed for metal (Cu and Zn) and nutrient (total phosphorous and nitrogen) concentrations. All life-history endpoints were negatively affected by the exposure, with higher mortality, reduced growth and lower fecundity in the harbours compared to the reference sites. Metal concentrations were the key explanatory variables for all observed adverse effects, suggesting that metal-driven toxicity, which is likely to stem from AF paints, is a source of anthropogenic stress for biota in the harbours.

## Introduction

Pollution caused by boating activities is a well-known problem, largely due to the use of antifouling (AF) paints [1–4]. In the Baltic Sea, the most commonly used AF paints contain metals, such as copper (Cu) and zinc (Zn), whereas organic biocides are forbidden [5]. The number of leisure boats in the Baltic Sea is approximately 2 million, with half of them located in Sweden [6]. Leisure boats are stationary 90% of the time [7], leaching biocides and contributing to increased pollution in harbours [8]. In Sweden, the input of Cu from AF paints into surface waters was estimated 104 tonnes/year, which is twice the input from forest land runoff and 7-fold the input from atmospheric deposition. This makes AF paints the main diffuse source of Cu in the surface waters [9]. Elevated Cu and Zn levels in the sediment have been linked to AF paint use in several harbours on the west coast of Sweden, including natural harbours situated in pristine areas [10].

Antifouling paints are designed to have a toxic effect on the biofouling organisms but release biocides, which are also toxic to non-target organisms. Several laboratory studies have reported toxic effects of AF paint leachates, such as growth inhibition of algae and mortality in copepods [11,12], while field evidence is scarce. It is important to assess the effects of the AF substances in the aquatic environment, especially when the active ingredients, i.e. metals, are not biodegradable, and will, therefore, be persistent. Moreover, investigating the interaction effects of metals and other abiotic factors commonly varying in estuarine habitats, e.g., nutrients and salinity, is highly relevant for improving prediction of toxic effects.

Long-term studies facilitate risk assessment at the population level and provide an understanding of the sublethal effects of pollutants. Field experiments (e.g., using caging technique) allow experimentation under natural conditions and, hence, higher external validity, compared to the laboratory experiments [13]. Long-term field experiments integrate responses to both natural variability in the environmental factors (e.g., temperature and salinity) and environmentally relevant levels of contaminants to which the organisms are exposed. Also, some endpoints, such as reproduction, can in some cases only be assessed in the natural environment, because some species mate poorly under laboratory conditions [14].

The use of molecular biomarkers as functional measures of exposure to contaminants is increasingly being adopted in risk assessment [13,15,16]. In particular, the induction of metallothionein (MT) or metallothionein-like proteins (MTLP) has been commonly used to detect exposure to both essential, e.g. Cu and Zn, as well as non-essential metals, e.g. Cd and Ag [17,18]. Metallothioneins are non-enzymatic proteins with high cysteine content which bind heavy metals thereby playing an important role in their detoxification [19,20]. Therefore, elevated MTLP concentrations are generally observed in organisms collected from metal-contaminated sites [21,22].

While effects of AF substances on target species have been evaluated, their potential impacts on non-target species are poorly known. Clearly, understanding these impacts for ecologically relevant species is a prerequisite for environmental risk assessment and protection of ecosystem integrity. Gastropods are the taxonomically largest group of marine animals, ~40 000 species [23]. Thus, they constitute a substantial part of the biodiversity; they also perform essential ecological functions in the aquatic systems, both as grazers and as prey for higher trophic levels. In general, snails have limited capacity to metabolize contaminants and can, therefore, bioaccumulate them to a greater extent than other animals [24]. In addition to the direct toxic effect (e.g. mortality), contaminants can also weaken the immune system of the snails, rendering them more susceptible to pathogen infections [25], which might ultimately affect reproduction and survival. During the reproductive season, molluscs are particularly sensitive to stress (e.g. parasites, thermal stress, contaminants) due to the high energy cost of spawning [26]. In marinas, all these multiple stressors are likely to interact with each other and to increase the chances of population decline for this ecologically important functional group [27].

Here, we use the nerite snail *Theodoxus fluviatilis* as a model organism. It is the second most widespread snail in the Baltic Sea [28] and is also commonly found in European freshwaters [29]. This snail is a generalist grazer, feeding on epilithic algae and detritus, and has a lifespan of 2–3 years [14]. In this study, we focused on effects on life history responses in *T*. *fluviatilis* induced by exposure to harbour contaminants. Snails were collected at a pristine site in the Baltic Sea and caged at several harbours and reference areas for 8–16 weeks. Mortality, growth, and reproduction, which are highly ecologically relevant endpoints, were analysed. In addition, snail MTLP levels were analysed and linked to metal concentrations in water and sediments at the exposure sites. We hypothesized that animals caged in the harbours would have higher mortality, lower growth, and lower fecundity, compared to those exposed at the reference sites (Hypothesis 1), and that these effects will be correlated to both the dissolved and sediment levels of Cu and Zn, the active substances in AF paints (Hypothesis 2). We also expected levels of MTLP to increase with increasing Cu and Zn concentrations in the environment (Hypothesis 3). Moreover, we evaluated the role of other environmental factors (nutrient availability, salinity, and pH) in modulating effects of Cu and Zn on the snails.

## Materials and methods

### Study locations

**Fig 1:**
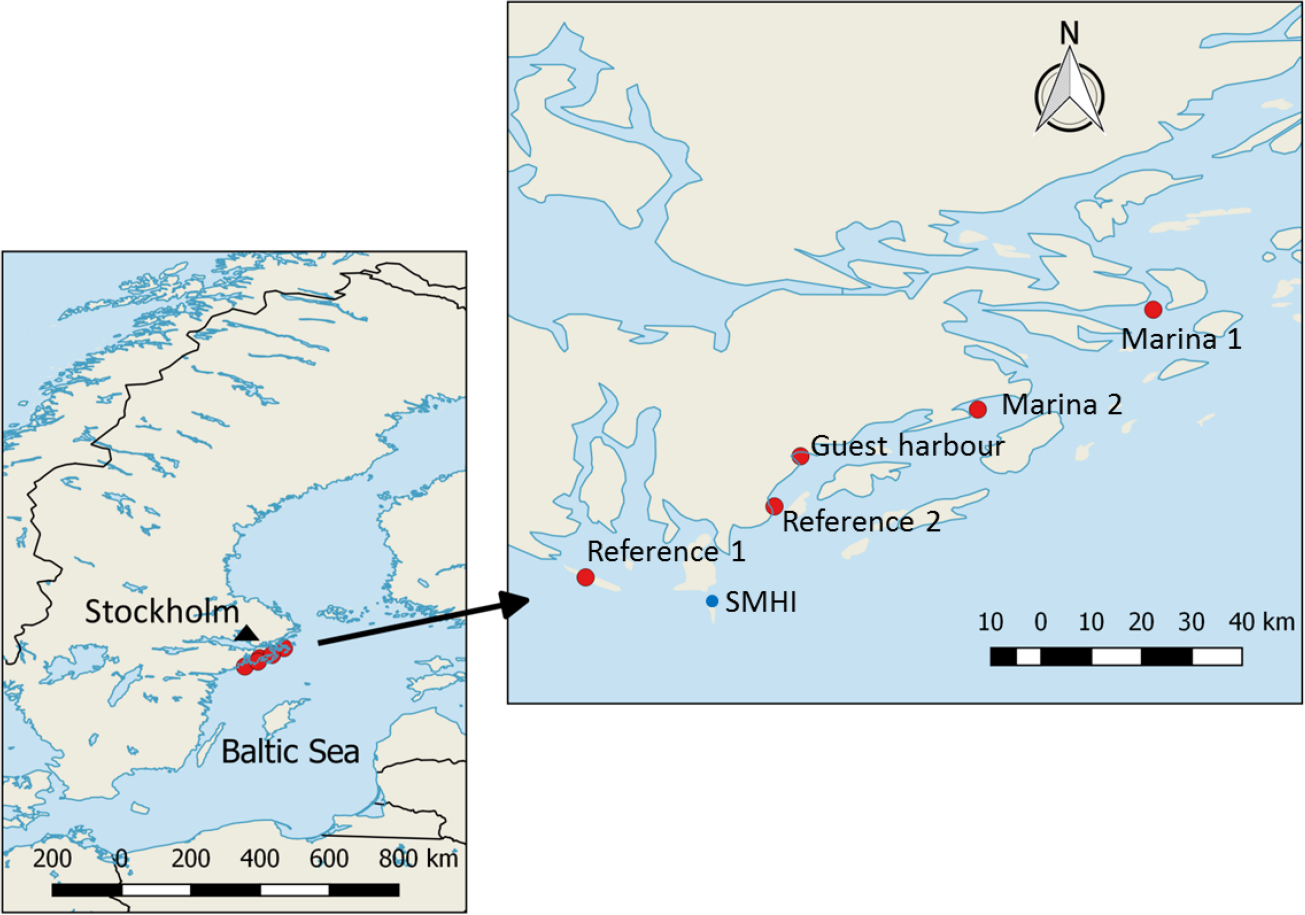
Map of the study locations and SMHI (Swedish Meteorological and Hydrological Institute) station that was used to retrieve the temperature data.

Permission for carrying out the experiments was obtained verbally from the respective harbour masters. Two experiments were conducted over two consecutive years: 2014 (*year 1*) and 2015 (*year 2*). In year 1, a commercial marina (*Marina 1*) and a guest harbour were chosen as representative areas with relatively high levels of contamination originating from AF substances. The main differences between marinas and guest harbours are outlined in Table 1. No industrial sites, sewage treatment plant effluents or other point sources of pollution were identified in the nearby areas. The reference site (*Reference 1*) was a pristine area located 80 km south of Stockholm and not directly exposed to any sources of AF paints. In year 2, the study was expanded by including an additional marina (*Marina 2*), increasing the number of snails per cage (in order to have enough material for biomarker analysis), and increasing the exposure time. Moreover, a new and easily accessible reference site was chosen (*Reference 2*). The three harbours included in the study operated 900 to 1 400 boats per year and were located within 40–80 km of Stockholm, Sweden (Fig 1). The depth at each location was estimated from nautical maps (Table 1). For simplicity, Marina 1 and 2 and the Guest Harbour are called ‘harbours’ when making general statements throughout the paper.

**Table 1:** Details on boating activities at the study locations.

Site	Residence time of boats	Boat maintenance activities	Depth (m)
Marina 1	long (entire season)	frequent	~3 to > 6
Marina 2			~6
Guest harbour	short (mainly visiting boats)	rare	8–11
Reference 1	no boats	none	~3
Reference 2			~3

#### Water and sediment sampling

Water samples were collected from 1 m depth using a Ruttner sampler and sediment was collected with an Ekman grab sampler; all the samples were placed in acid-washed plastic containers, transported to the laboratory in cooling boxes and stored at 4°C until analysis (ca 4 months). Approximately 2 cm from the top sediment were used for the chemical analysis. The samples were collected at the start of the experiment.

#### Physicochemical parameters

Measurements of pH and salinity were performed in the laboratory using Metter Toledo FiveEasy™ FE20 and FG3, respectively. Data on surface water temperature were collected by the Swedish Meteorological and Hydrological Institute (SMHI) at the nearby monitoring station (58°46'08.0"N 17°51'32.0"E; Fig 1). The temperature data were retrieved from the SMHI database (http://sharkdata.se) and average temperatures were calculated for the exposure period of our experiment. Nutrient concentrations (nitrogen and phosphorus) were measured in the water samples from each location. Total nitrogen (TN) and phosphorus (TP) were analysed using Autoanalyzer II (Technicon), according to the standards SS-EN ISO 11905–1:1998 and SS-EN ISO 15681–2:2005, respectively.

### Experimental design and test animals

In both years, the experimental animals were collected at the site Reference 1 to minimize genetic variability among individuals, and to ensure that snails had similar physiological conditions prior to the exposure. There was no pre-acclimatisation of the animals to the experimental environments. *T*. *fluviatilis* is an invertebrate and a species of Least Concern on the IUCN (International Union for Conservation of Nature) Red List of Threatened Species™ and hence no ethical permit was required for conducting this study.

Plastic 100-mL cages (S1 Fig) were used for *in situ* exposure. Each cage contained 5 or 10 snails (in year 1 and 2, respectively) of different sizes (3 to 8 mm shell length) and fresh tips of bladderwrack (*Fucus vesiculosus*) as a natural substrate, collected from Reference 1. During the exposure, the snails were also able to graze on the ambient periphyton growing inside the cages. Thirty cages were placed at each site, tied to the docks in the harbours or to the buoys at the reference sites at 1 m beneath the water surface. The snails were not sexed before the caging; however, in *T*. *fluviatilis* the sexes are separate, with a sex ratio of ~1:1 in the Baltic Sea [14], which means that each cage was likely to contain both males and females. Females lay 20–40 egg capsules of ca 1 mm diameter, which are attached to the substrate. Each capsule contains ca 80–100 eggs, out of which only one juvenile hatches and has the morphology of a miniature adult (i.e. there is no free-swimming larval stage). Egg-laying occurs throughout the year, but most abundantly in the summer [30].

The exposure time was defined as the time spent in the cage in the test area. The exposure lasted eight weeks in year 1 (mid-July to mid-September) and 16 weeks in year 2 (end of May to mid-September), thus covering most of the boating season in the area (May to early October). During the exposure, the cages were periodically cleaned from biofouling to facilitate water exchange, and the bladderwrack was changed when necessary. Snail mortality was recorded at each field visit (e.g., every 2–4 weeks) and fecundity was assessed by counting the number of egg capsules in each cage after the first 2 or 4–5 weeks of exposure (year 1 and 2, respectively). The data are presented as mortality rates (Eq 1) and weight-specific fecundity (Eq 2). Growth was measured as an increase in the whole body mass (i.e. soft tissue and shell) over the entire exposure period and calculated as relative growth rate (RGR) using Eq 3. Details on the weight measurements are found in the supplementary material (S1 Text).

$$Mortality\;rate=\frac{N_{deadt}}{N_{alive0}}\frac{1}{t},$$ (Eq 1)

where N_dead_ is the number of snails that died by the end of the experiment (time *t*, 8 or 16 weeks for year 1 and year 2, respectively) and N_alive_ is the number of snails at the start, time *0*.

$$Fecundity\;rate=\frac{N_{eggst}}{W_{0}}\frac{1}{t},$$ (Eq 2)

where N_eggs_ is the number of egg capsules at time t (2 and 4–5 weeks for year 1 and 2, respectively) and W_0_ is the weight of the snails at the start (mg dw).

$$RGR=\frac{\ln\;(W_{t})-\ln\;(W_{0})}{t},$$ (Eq 3)

where W_t_ is the dry weight of the snails at the end of the experiment.

### Analysis of metals and organic antifouling biocides

Concentrations of dissolved metals (Cu and Zn) were measured in the water samples from each location. Cu and Zn were also analysed in the sediment samples from each location (SS EN ISO 17294–1 mod). In addition, irgarol, diuron and organotins MBT, DBT and TBT (mono-, di- and tributyl tin, respectively) were measured in the sediments (year 1 only). The analysis of organic biocides was carried out by a certified laboratory (ALS Scandinavia, according to DIN ISO 38407–35) and dissolved metals were analysed at both ALS (SS EN ISO 17294–1 mod) and Stockholm University (SS EN ISO 17294–2:2005).

### Analysis of metallothionein-like proteins

At the end of the exposure period, the surviving snails were frozen in liquid nitrogen and stored at -80°C until analysis. A spectrophotometric method modified from [31] was used for quantifying MTLP in the soft tissues. The whole shell-free body was used for the analysis as it has been shown to be more reliable for long-term monitoring than dissected digestive glands [32]. The tissues of 3–6 snails were pooled into a sample (50.4 ± 0.2 mg wet weight; 12–18 samples per site) and homogenized with Buffer 1 (1:3) containing 0.5 M sucrose, 20 mM Tris-HCl buffer (pH 8.6), 0.006 mM leupeptin, 0.5 mM phenylmethylsulphonilfluoride (PMSF) and 0.01% β-mercaptoethanol. The homogenization was done using FastPrep®-24 (MP Biomedicals), ~34 mg zirconium/silica beads (0.5 mm, BioSpec) and one ceramic sphere (6.35 mm, MP Biomedicals) at a speed of 6 m/s for five cycles of 20 sec each (with cooling on ice in-between). The homogenates were further centrifuged at 30 000 g for 30 min, and 100 μL of the supernatant were used for fractionation of MTLP with cold absolute ethanol (101 μL,—20°C) and chloroform (8 μL). The samples were centrifuged at 6 000 g for 10 min, and 180 μL of the supernatant were combined with 1 mg RNA, 4 μL 37% HCl and 3 volumes of cold, absolute ethanol and kept at -20°C for 1.5 h for precipitation of MTLP. The samples were then centrifuged at 6 000 g for 10 min, after which the MTLP-containing pellet was washed with 87:1:12 ethanol, chloroform and Buffer 2 (prepared as Buffer 1 but without leupeptin, PMSF and β-mercaptoethanol) and centrifuged again at 6 000 g for 10 min. The supernatant was discarded, and the pellets were dried under N gas and re-suspended in Buffer 3 (50 μL 0.25 M NaCl, 50 μL 1N HCl and 4 mM EDTA from Merck). To each sample, 800 μL of the reagent solution containing 2 M NaCl, 0.2 M Na-phosphate pH 8 and 0.43 mM DTNB (5,5-dithiobis-2-nitrobenzoic acid) were added, followed by centrifugation at 3 000 g for 5 min. The absorbance of the supernatant at 412 nm was measured with a spectrophotometer (Shimadzu UV-2501PC) using reduced glutathione as standard. All centrifugation steps were conducted at 4°C, with the exception of the last one that took place at room temperature. The amount of MTLP in the snails was calculated assuming a cysteine content of 18% [33]. Information regarding recovery rates and detection limits is found in the supplementary material (S2 Text). All reagents were of analytical grade and purchased from Sigma Aldrich or VWR unless stated otherwise.

### Data analysis

**Fig 2:**
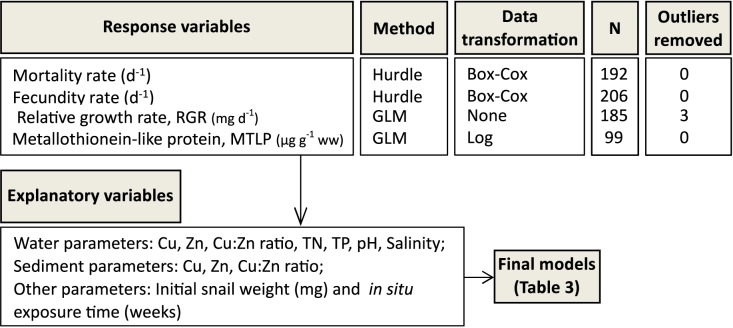
Response and explanatory variables considered in the regression models. TP = total phosphorous, TN = total nitrogen, GLM = generalized linear model, ww = wet weight.

First, the variability of the life-history and abiotic variables within the dataset was explored using between-group PCA (bgPCA) plots as implemented in PAST software v. 3.14. All other statistical analyses were conducted in JMP software v. 12 (SAS, USA). Generalized linear models (GLM) were used to identify the abiotic factors (concentrations of metals, TN, TP, salinity, pH and the relevant interactions) affecting responses in the snails (growth, fecundity, MTLP and mortality). In addition to the metal concentrations, Cu:Zn ratios for dissolved metals and those measured in the sediment samples were also used in the GLMs as predictors to investigate potential mixture effects [34]; the effect was interpreted as antagonistic when the metal ratio predicted a positive (beneficial) biological effect and synergistic when it was negative (adverse)(S1 Table). The growth model also included the size of the snails at the beginning of the exposure, because in these animals growth rates are size-specific [35]. The summary of the dependent and independent variables used in different models is shown in Fig 2. For evaluating mortality and fecundity rates that were zero-inflated, we used the Hurdle model [36] with two submodels: 1) a binomial logistic regression predicting the probability of zero mortality and zero fecundity, respectively; 2) GLM on zero-truncated mortality and fecundity, using a normal error structure and log link function. The model evaluation was done by assessing normality and homogeneity of variance in residual plots. The zero-truncated mortality and fecundity data were Box-Cox transformed to achieve a normal distribution. Outliers in the growth data were identified with Grubb’s test and removed from the analysis; this procedure did not affect the significance of the predictors but improved the normality of the residuals. The criteria for choosing the best model were based on the AIC value and the parsimony of the models. To observe the changes in mortality over time, standard survival curves were fitted for each location. For this, individuals who were lost or alive at the end of the exposure period were censored, and log-rank tests were used to check for differences in survivorship between the sites. Differences between site types (harbours vs. references) in fecundity, growth and MTLP were also tested using GLMs. Furthermore, because MTLP levels may be affected by physiological changes in the organism [37], Spearman correlation was used for investigating potential relationships between MTLP and growth-related variables (RGR and fecundity).

## Results

### Between-site variability in environmental conditions and life-history responses

In both years, the harbours differed substantially from the reference sites in terms of the direction and variability of life history responses and environmental conditions. High variability was also observed between the harbours within a year (S3 Fig). Nevertheless, in both years, the reference sites were associated with high fecundity and high nutrient levels (TN and TP in year 1 and TN in year 2). In contrast to the reference sites, the harbour environments were associated with elevated concentrations of metals, either dissolved (Marina 1) or associated with the sediment (Guest harbour in year 1 and Marina 2) as well as high mortality and low RGR (Marina 1) and low fecundity (Marina 2) of the snails. Moreover, low salinity and relatively low pH contributed to the separation of Marina 1, whereas high TP and low TN levels contributed to the separation of Guest harbour in year 2. Thus, the studied environments showed a high variability in the environmental factors that was both related and unrelated to the metal contamination.

### Metals and organic antifouling biocides

There were higher concentrations of dissolved Cu (2.7–3.7 μg/L) in both marinas compared to the reference sites (0.6–1.2 μg/L), whereas intermediate levels were observed in the Guest harbour (1.4–1.8 μg/L). Likewise, dissolved Zn concentrations in the marinas (7.1–10.6 μg/L) were approximately 3-fold higher than those observed at Reference 2. Similarly, the sediments in the harbours had higher concentrations of metals and organic tin compounds, compared to the reference sites, except for the sediment from Reference 1. The levels of Cu and Zn in the harbour sediments varied 23–61 and 65–116 mg/kg TS (total solids), respectively, exceeding those at the reference sites by up to a factor of 11 for Cu and 7 for Zn. The concentrations of organotin compounds at Reference 1 and Marina 1 were comparable to the low levels previously reported in reference areas of the Baltic Sea (e.g., 2–10 μg TBT/kg TS)[38], whereas the levels in the Guest harbour were similar to previous reports from other harbours in the Stockholm archipelago [38,39]. Irgarol and diuron were below detection limit at all sites (Table 2).

**Table 2:** Metals and organic antifouling biocides in sediment (s) and water (w) from harbours and reference sites; TS = total solids, TBT, DBT and MBT = tri, di and monobutyl tin, respectively; TN = total nitrogen, TP = total phosphorous; one sediment sample was analysed per site Concentrations in water are in μg/L and in sediment in mg/kg TS and μg/kg TS for organotins.

Parameter	Year	Guest harbour		Marina 1		Marina 2		Reference 1		Reference 2	
		s	w	s	w	s	w	s	w	s	w
Total solids (TS, %)	2014	45.7		59.9				76.8			
	2015	56.2		36.7		51.2				88.4	
Cu	2014	59.6	1.4	37	3.7			126	1.2		
	2015	33.4	1.8	61.2	3.5	23	2.7			5.5	0.6
Zn	2014	116	1.8	64.8	7.1			67.1	7.4		
	2015	101	3.3	162	10.6	69.2	8.1			16.6	3.4
TBT	2014	86		26.6				0.91			
DBT	2014	58.1		8.86				<1			
MBT	2014	46.9		19.3				1.29			
Irgarol	2014	<0.01		<0.01				<0.01			
Diuron	2014	<0.01		<0.01				<0.01			
TN	2014		295		308				338		
	2015		268		288		288				300
TP	2014		16		15				24		
	2015		18		12		16				10

### Physicochemical parameters

The nutrient levels varied 288–338 μg/L for TN and 10–24 μg/L for TP (Table 2). The TN levels were very similar between harbours and reference areas for both years, whereas TP was 1.6-fold lower and 1.8-fold higher in the harbours than at reference sites in year 1 and 2, respectively. The salinity varied between 5.47–5.80 PSU at the reference sites and 4.82–5.75 PSU in the harbours, thus not differing by more than 1.2-fold between the two types of locations. The pH was very similar for the reference sites and harbours, ranging between 7.94–8.34 and 7.91–7.98, respectively. The surface water temperature was higher in year 1 than in year 2 (18.0 and 15.1°C, respectively, S2 Fig).

### Mortality

**Fig 3:**
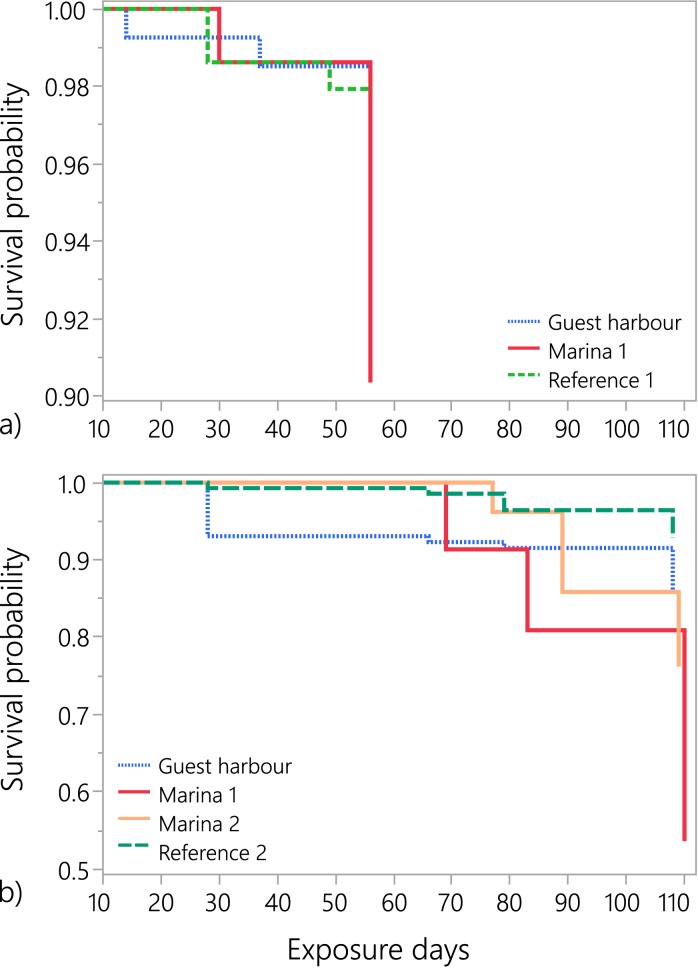
Survival curves for snails exposed at different locations in year 1 (a) and 2 (b); note the different scales on the y-axis.

**Fig 4:**
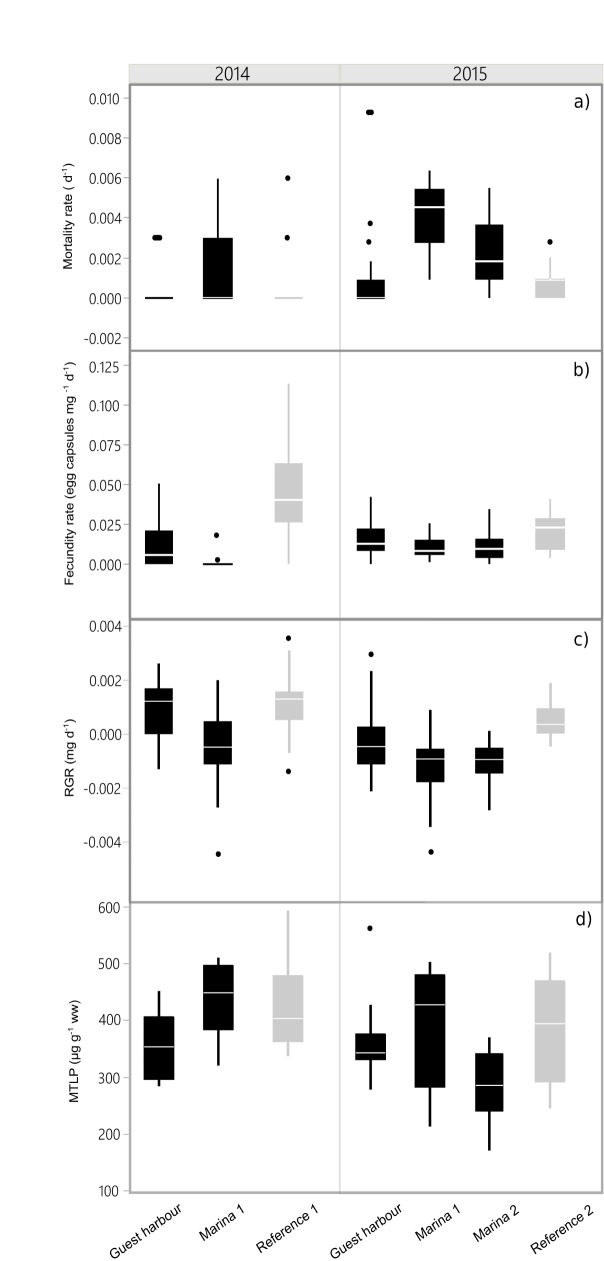
Mortality and sublethal responses of snails exposed in different locations. For each variable, the horizontal line indicates the median, the lower and upper limits of the boxes are the 1^st^ and 3^rd^ quartile, respectively and the whiskers show the furthest point within 1.5 × the interquartile range. Harbours and reference sites are shown in black and grey, respectively. MTLP = metallothionein-like proteins, RGR = relative growth rate.

During both years, the snail mortality rate was higher in the two marinas compared to the reference areas (Figs 3 and 4A). In general, the survival of the snails in the marinas decreased by up to 7% per week, compared to the reference sites where the survival decreased by maximum 1% per week (Fig 3; log-rank p = 0.006 for year 1 and 0.003 for year 2). The probability of survival was significantly higher at low concentration of dissolved Zn, yet relatively high concentration of dissolved Cu; the survival was also positively related to Cu:Zn ratio in sediment, indicating antagonistic interactions between the two metals. Moreover, Cu concentrations in water and sediment were the best positive predictors of mortality rate, with dissolved Cu being a stronger predictor than Cu_sed_ (Table 3A).

**Table 3:** Regression models testing effects of environmental variables on snail life-history and biomarker responses: a) Hurdle models for zero-inflated data (mortality and fecundity rate); b) Generalized linear models (GLMs) for the growth (RGR) and MTLP responses.

Effect	Predictors	Estimate	SE	Chi^2^	p
a) Hurdle models					
***Mortality***					
1) Zero mortality submodel (probability of survival)	Cu	8.45E-01	2.57E-01	10.79	0.001
	Zn	-7.84E-01	1.44E-01	29.74	< .0001
	Cu_sed_:Zn_sed_	3.85E+00	6.17E-01	38.89	< .0001
2) Positive mortality submodel	Cu	1.67E-02	5.27E-03	9.57	0.002
	Cu_sed_	9.43E-04	2.39E-04	14.43	0.0001
***Fecundity***					
1) Zero fecundity submodel (probability of failed reproduction)	Cu	5.26E+00	8.14E-01	41.64	< .0001
	salinity	1.10E+01	1.95E+00	32.09	< .0001
2) Positive fecundity submodel	Cu	-5.20E-02	1.13E-02	20.04	< .0001
** **	Cu_sed_:Zn_sed_	1.81E-01	1.94E-02	69.72	< .0001
b) GLMs					
***RGR***	Zn	-2.38E-04	3.00E-05	54.11	< .0001
	Cu_sed_:Zn_sed_	2.30E-03	3.00E-04	51.27	< .0001
	Initial weight	-1.78E-04	4.60E-05	14.37	0.0002
***MTLP***	Zn	-9.57E-02	2.83E-02	12.32	0.0004
	Cu_sed_:Zn_sed_	4.67E-01	1.09E-01	19.74	< .0001
	salinity	-6.95E-01	1.90E-01	14.25	0.0002

### Sublethal effects

#### Growth

The growth rates were lower in the animals exposed in the harbours, compared to those from the reference sites (p = 0.0016 and p < 0.0001 for years 1 and 2, respectively; Fig 4C). Negative growth was observed in the harbours (except Guest harbour in year 1), with average mass decrease of 3 and 11% (year 1 and 2, respectively), whereas growth at the reference sites was positive, with average 5 and 9% in year 1 and 2, respectively. The lowest growth rates were observed in Marina 1 during both years (Fig 4C). As expected, the growth was size-dependent, with initial body weight having a significant negative effect on RGR. Moreover, the RGR was negatively associated with dissolved Zn and positively with the Cu:Zn ratio in the sediment, indicating a potential antagonistic effect of the two metals on snail growth (Table 3B).

#### Fecundity

In both years, the fecundity rate was significantly lower in the snails exposed in the harbours, compared to those from the reference areas (p < 0.0001 for both years; Fig 4B). In particular, Marina 1 was the site with almost no fecundity in year 1. Only 2 out of 30 cages contained egg capsules, which were present in small numbers; thus, the fecundity rate at this site was 67-fold lower than at Reference 1. Across the sites, fecundity was negatively affected by dissolved Cu and salinity (Table 3A). Moreover, Cu:Zn ratio in the sediment was a significant positive predictor, indicating a possible antagonistic effect of the two metals on snail reproduction.

#### Metallothionein

The MTLP levels in the harbour-exposed snails were not significantly different from those from the reference sites (p = 0.192 and 0.255 for years 1 and 2; Fig 4D). Nonetheless, the highest MTLP levels were observed at Marina 1 in both years. The GLM identified dissolved Zn, salinity, Cu:Zn ratio in the sediment as significant predictors of MTLP (Table 3B). The Cu:Zn ratio was positively associated with MTLP, suggesting potential synergistic effect between the two metals. The MTLP levels decreased with salinity and increasing levels of dissolved Zn. No significant correlation was found between MTLP and fecundity (p = 0.76) or growth (p = 0.818).

## Discussion

Although there was a high variability of physiological responses and environments among all study sites, there were higher metal concentrations in the harbours compared to the reference sites and lower fitness of the snails caged in the harbours. We found that Cu and Zn, i.e., the metals originating to a great extent from AF paints, were the key predictors of the observed biological responses. Therefore, the Hypotheses 1 and 2 were supported, and the harbour environments, boating activities and AF paints were identified as significant contributors to the suboptimal environment for *T*. *fluviatilis*. The poorest survival, growth, and reproduction of the snails, as well as highest MTLP levels, were observed at the site Marina 1, one of the largest marinas for recreational boats in the Baltic Sea; thus, it is not surprising that snails in this habitat were the most heavily impacted.

### High metal concentrations in the harbours

The harbours investigated in this study had sediment Cu and Zn levels similar to other marinas around the world, e.g., Cu: 16 μg/g TS [40], 63 mg/kg TS [41] and 210 mg/kg [42], Zn: 94 mg/kg TS [41] and 53 mg/kg TS [40]. Reference 1 also had high levels of Cu in the sediment, which was unexpected, since the values previously reported for that site were 4-fold lower [43]. Furthermore, the dissolved Cu concentrations in the harbour waters were comparable to the values reported in harbour areas, e.g., San Diego Bay: 5–6 μg/L [42,44]; Bahia Blanca Estuary: 5 μg/L [40]. At our reference sites, dissolved Cu and Zn concentrations were similar or slightly higher compared to the background levels estimated for the Baltic Sea (0.3 and 1 μg/L for Cu and Zn, respectively) [45], whereas in the harbours, the metal concentrations exceeded these levels up to 12- and 11-fold for Cu and Zn, respectively (Table 2). Thus, the metal concentrations in the harbours substantially exceeded the target values for Good Environmental Status in the Baltic Sea (1.45 and 1.1 μg/L for Cu and Zn, respectively) [46]. Since no major point sources of Cu and Zn emissions were located in the vicinity of the study sites, we believe that the levels of these metals reflect inputs from AF paints used on leisure boat hulls. Several studies have also shown a metal release from AF paints in marinas [8,47,48] Such elevated metal concentrations can impact various species inhabiting harbour areas, including snails [49,50].

### Variability of the physicochemical factors

In our study, total nitrogen and phosphorous were used as measures of the nutritional conditions in the studied areas. The values recorded (TN: 297.9 ± 21.7 μg/L and TP: 15.9 ± 4.5 μg/L; Table 2), were similar to the nutrient levels in the Baltic Sea during summer. For example, SMHI (http://sharkdata.se station B1 in the northern Baltic proper, 58°48'00.0"N 17°37'00.1"E) reports TN and TP in the surface water during May-September to be 307.8 ± 41.3 μg/L and 22.2 ± 2.5 μg/L, respectively. In general, nutrient deficiency can cause growth inhibition both directly by energy limitation or indirectly by rendering the test animals more sensitive to pollutants and other stressors [51]. In this study, we did not observe any effects of nutrients on the life-history or MTLP levels of the snails. Moreover, we have not detected any statistically significant interaction between metals and nutrients, while other studies [52] showed that antagonistic interactions might occur. The lack of such effects in our study can be related to the relatively low variability of the nutrient concentrations. Besides the nutrients, the salinity and pH were also very similar between harbours and reference sites. This implies that more locations with controlled gradients should be employed in field studies to adequately assess the effects of multiple stressors (e.g. nutrient abundance or limitation, salinity and pH fluctuations, among others) on environmentally relevant species and their responses to contaminants.

Variability in the summer temperature may have contributed to the between-year differences in growth and fecundity observed in our study. The average temperature in the study area during the experiment was 18.0 and 15.1°C for year 1 and 2, respectively (S2 Fig), which may explain the higher growth and fecundity in year 1. As temperature is an important factor initiating egg-laying in *T*. *fluviatilis* [30], the between-year difference in the average water temperature during the first half of the exposure (20.8°C vs. 12.7°C) was sufficient to induce the observed difference in reproductive output. It is also possible that the lower density of animals in the cages during year 1, and hence lower competition for food, might have contributed to higher growth and reproduction during this experiment.

### Elevated Cu and Zn levels as major drivers of fitness reduction

Environmental metal concentrations in the harbours were found to negatively affect the main fitness parameters (survivorship, growth and reproduction) in the snails (Fig 4). Indeed, all these endpoints were significantly affected by metals, particularly Cu (Table 3). The observed mortality did not exceed 50% at the maximum concentrations of 3.7 μg/L and 10.6 μg/L for Cu and Zn, respectively, which is in line with the published toxicity data for various snail taxa, where chronic (28 d) exposure resulted in LC_50_ varying between 13 and 42 μg/L for Cu [53]. Similar mortality response was also observed in the snail *Biomphalaria glabrata* (Heterobranchia, Planorbidae that had 20% mortality when exposed for 33 d to 15 μg/L Zn [54].

In the harbour-exposed snails, mortality increased by up to 7-fold, in concert with growth becoming negative and reproduction declining up to 67-fold. The latter could have been affected both directly, as a result of reproductive toxicity, and indirectly, as a result of the inhibition of somatic growth. The reduced growth in harbours is in line with other reports, e.g., a reduction in growth for *Lymnaea stagnalis* at Cu levels similar to Marina 1 [55] and Cu-induced growth reduction in several species of snails, with EC_20_ ranging from 8.2 to 27 μg/L [53]. It is possible that, due to the higher levels of contaminants in the harbours, the snails employed coping mechanisms that were energetically costly and led to the negative energy budget and losses of body mass.

The lower fecundity rates associated with dissolved Cu concentration (Table 3A) are also in line with observations for other freshwater snails, e.g. *Stagnicola vulnerata* [56], *Lymnaea stagnalis* [55], though at higher Cu concentrations. Moreover, we observed a positive effect of the sediment Cu:Zn ratio on fecundity, growth and the probability of survival, indicating possible antagonistic effects between Cu and Zn. Similar interactions between the two metals were observed for the reproduction of *Ceriodaphnia dubia* [57]. Competition between Cu and Zn for binding at the target sites might explain the antagonistic effect of the mixtures [58]. The fact that metals in both dissolved form and from the sediment were important predictors of toxic responses suggests that the role of metals depends on complex-forming properties (e.g., hardness, redox potential, organic matter) [59] that were not measured in this study. The sediment metal ratios have implications for the long-term effects of AF paints in harbours, as metals will persist in the sediment even after their use in the paints is ceased.

Several studies on aquatic invertebrates have shown that dietary exposure is more important than waterborne exposure for metal bioaccumulation and toxicity [60,61]. We believe that was the case in our study as well, as the snails do not drink water, since they are osmoconformers. Hence, the ion pumps in the gills are not as active as in freshwater environments, which reduces active uptake of metal ions. Although metal concentrations in the periphyton growing in the cages were not measured, the fact that mortality increased over time in all harbours (Fig 3) suggests that snails accumulated pollutants from food, i.e., algal biofilms which can have a Cu bioaccumulation factor of up to 25 000 [62]. At the same time, the metals in the sediment also seem to play an important role for the observed effects. We hypothesize that the levels of metals in the sediment reflect the (unmeasured) levels of metals in the biofilm, as the two matrices have similar metal-binding characteristics [63]. This hypothesis is supported by several studies which have shown a strong positive correlation between metals in sediments and biofilm [63,64]. To identify the most important metal exposure pathways for non-target species with different feeding habits, future studies should employ carefully designed laboratory experiments in combination with field exposures.

### Metallothionein response: Importance of salinity and Cu:Zn ratio in sediment

Animals exposed at Marina 1 had the highest MTLP levels coinciding with the lowest survivorship, growth and reproduction. However, contrary to our hypothesis, there was no overall elevation in the MTLP levels in the snails exposed in the harbours, compared to those from the reference sites. The lack of response was, most probably, related to the confounding negative effects of salinity on the MTLP levels. A negative effect of salinity on MT was also observed in the gills of the crab *Pachygrapsus marmoratus* [65] and in the mussel *Mytilus galloprovincialis*, presumably due to changes in the general protein metabolism or due to shifts in the subcellular partitioning of metals [66]. Moreover, MT from *T*. *fluviatilis* has not been characterized yet and thus there are uncertainties regarding the response patterns of this protein upon exposure to Cu, Zn, and other unmeasured metals. For example, Cd has a higher binding affinity for MT compared to Zn [67], so Cd can replace Zn in MT, thus leading to transcriptional activation and MT upregulation. So Hypothesis 3 was not supported, and MTLP was not found to be a suitable biomarker for exposure to dissolved Cu and Zn in the harbour environments. However, the Cu:Zn ratio in sediment was a positive predictor for MTLP levels (Table 3B), which is a possible indication of synergistic effects between Cu and Zn. Both synergism and antagonism have been reported for Cu-Zn mixtures [68,69], which suggests that the type of interaction depends on the concentration of metals, the test species and the endpoints measured. Physiological changes associated with reproduction and growth have been found to increase the MTLP levels [37,51]; yet, we detected no statistically significant relationship between MTLP and fecundity or growth. The fact that the MTLP levels were not substantially higher in the harbours compared to the reference sites might also indicate that the snails exposed to high metal concentrations use metal-binding strategiesother than MT (e.g., lipofuscin) or granules composed of phosphates, oxalate and carbonates to store Cu and Zn in inert form [70].

The between-year MTLP variability was similar to that observed for growth and fecundity. The average MTLP concentrations were 14–16% higher in year 1 than in year 2. Both the difference in the water temperature and the exposure time could potentially contribute to that. Indeed, in other molluscs, the MTLP levels were found to be positively correlated to water temperature [37]. In order to fully evaluate the applicability of MTLP in the monitoring of biological effects of metal contaminants, the optimal response time of MTLP should be investigated.

## Conclusion

In conclusion, this study shows that leisure boat harbours accumulate metal contaminants and this has negative consequences for non-target species, such as the snail *T*. *fluviatilis*. As AF paints are one of the main sources of Cu in the studied areas, we ascribe the observed impacts to the metals leaching from boat coating. However, we did not analyse organic contaminants originating from e.g., oils used in motorboats and, therefore, their potential contribution to the observed toxic effects cannot be excluded. Further studies on metal bioaccumulation in periphyton and snails are needed in order to link the biological effects to the dietary exposure.

## Supporting information

S1 Table Mixture effects. (DOCX)

S1 Raw Data (XLSX)

S1 Fig Cages used for snail exposure. (TIFF)

S2 Fig Water temperatures. (TIFF)

S3 Fig Between-group PCA; a) year 1 and b) year 2.
(TIFF)

S1 Text Determination of snail dry weight. (DOCX)

S2 Text Quality control and calculation of metallothionein concentrations. (DOCX)

S3 Text Between-group PCA. (DOCX)
