# Supplementary material for: Metal contamination in harbours impacts life-history traits and metallothionein levels in snails
Source: PLoS One. 2017 Jul 3;12(7):e0180157. doi: 10.1371/journal.pone.0180157 (PMC5495383; doi:10.1371/journal.pone.0180157)
Supplement: S1 Table — (DOCX) [file pone.0180157.s001.docx]

**Mixture effects**

S1 Table. Direction of the relationship between biological effects and the metal ratios vs. the individual metal concentrations in the sediment, based on the GLM estimates.

|  | **Predictor variables** | | | |
| --- | --- | --- | --- | --- |
| **Response variable** | \| **Cu_sed_:Zn_sed_** \| \| --- \| | \| **Cu_sed_** \| \| --- \| | \| **Zn_sed_** \| \| --- \| | **Effect** |
| Fecundity rate | + | + | - | antagonism |
| Relative growth rate | + | + | - | antagonism |
| Metallothionein | + | + | n.s. | synergism* |

**synergism because an increase in MT is theoretically a sign of increased toxicity.*
