# Supplementary figures and images for: Metal contamination in harbours impacts life-history traits and metallothionein levels in snails
Source: PLoS One. 2017 Jul 3;12(7):e0180157. doi: 10.1371/journal.pone.0180157 (PMC5495383; doi:10.1371/journal.pone.0180157)

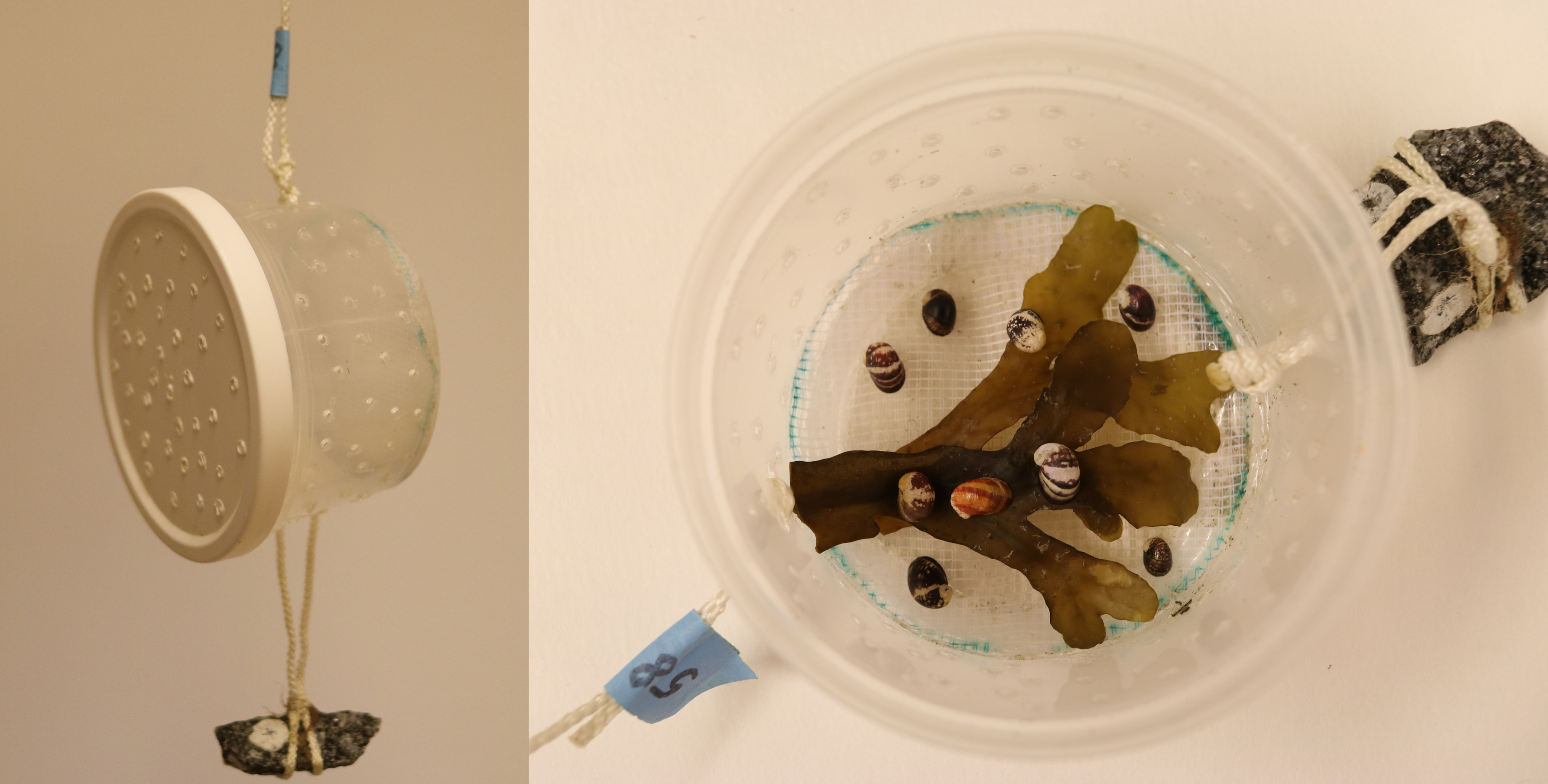

Supplement: S1 Fig — (TIFF) [file pone.0180157.s003.tiff]

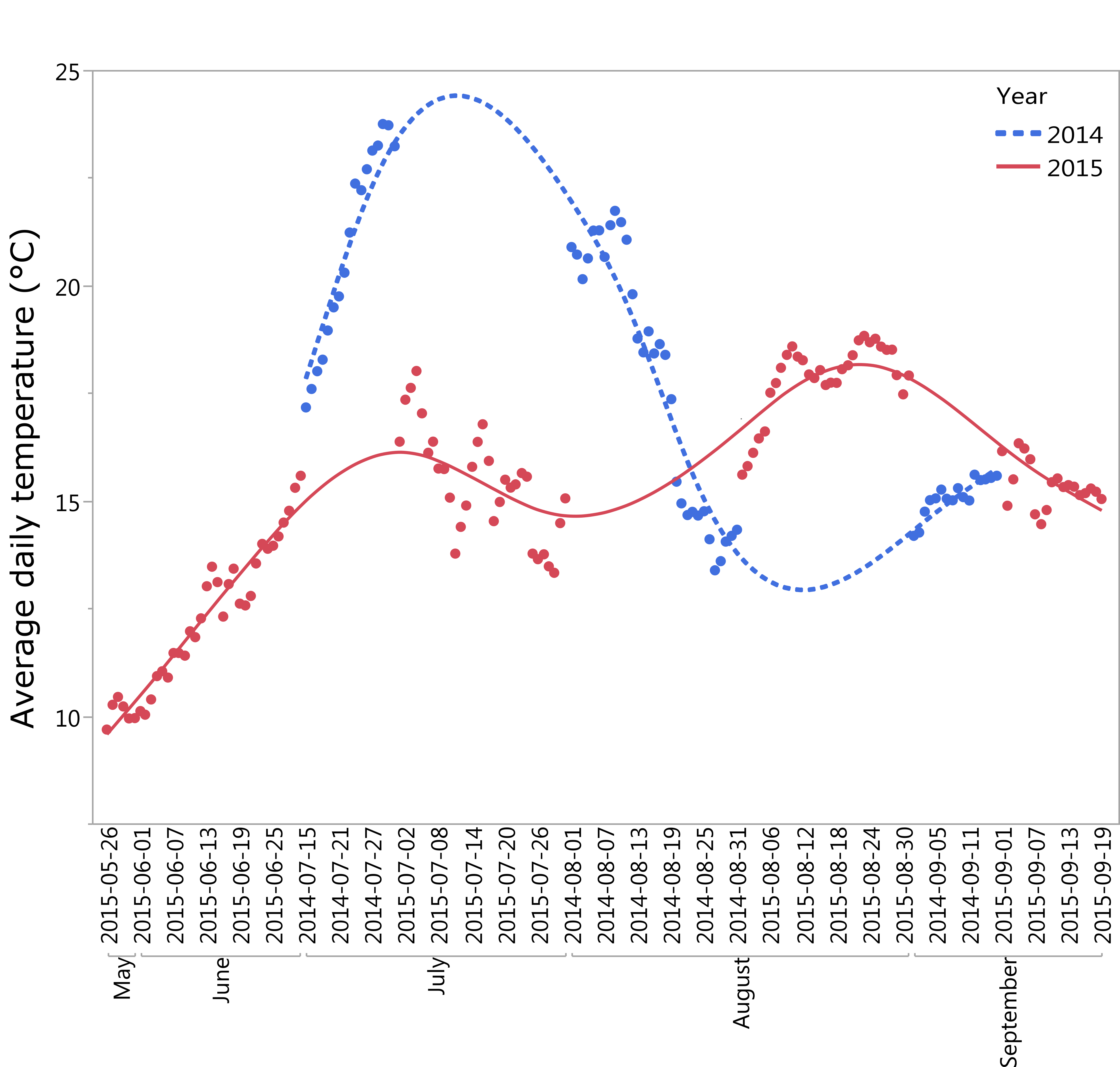

Supplement: S2 Fig — (TIFF) [file pone.0180157.s004.tiff]

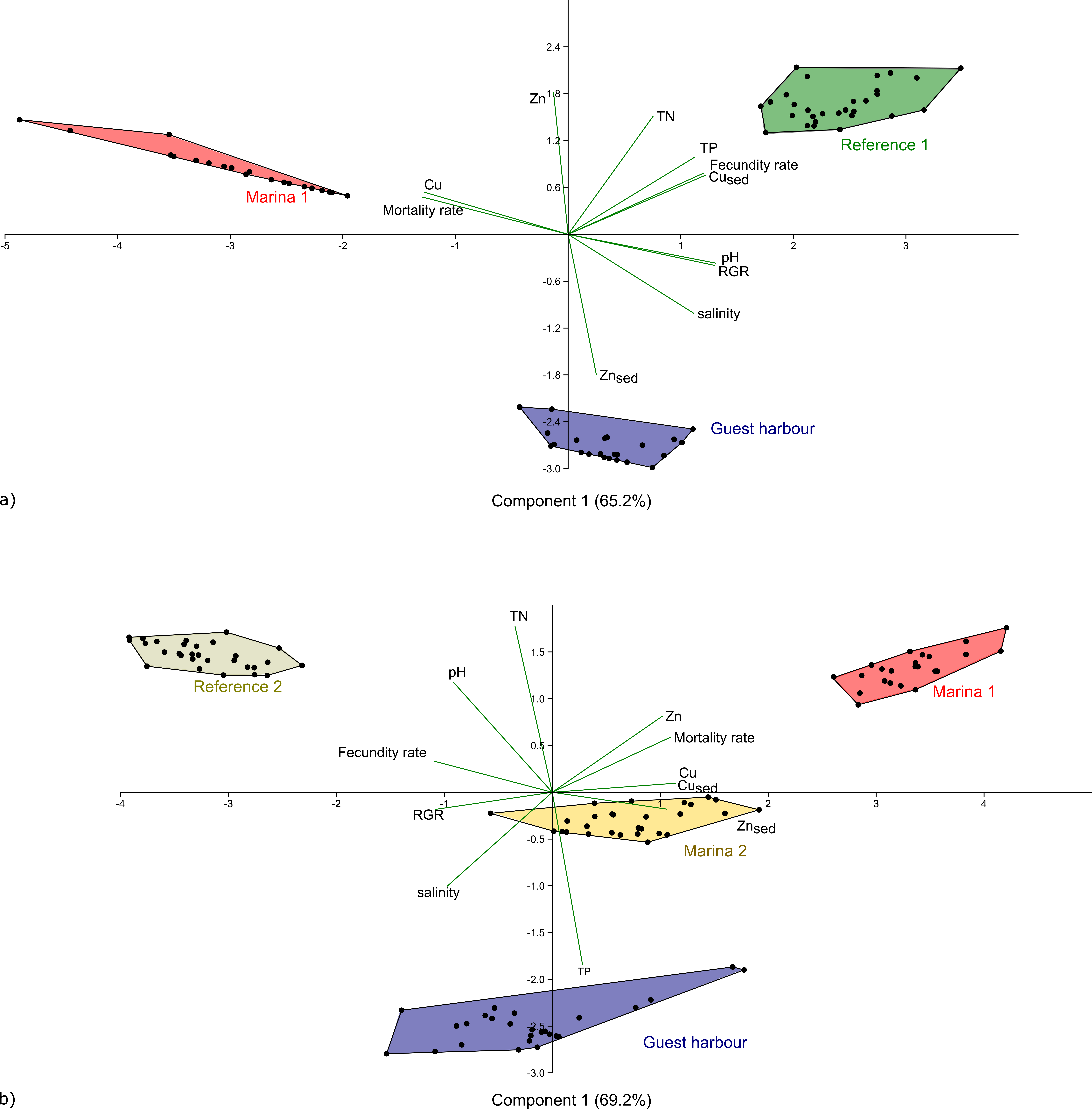

Supplement: S3 Fig — Between-group PCA; a) year 1 and b) year 2. (TIFF) [file pone.0180157.s005.tiff]
