# Supplementary material for: Metal contamination in harbours impacts life-history traits and metallothionein levels in snails
Source: PLoS One. 2017 Jul 3;12(7):e0180157. doi: 10.1371/journal.pone.0180157 (PMC5495383; doi:10.1371/journal.pone.0180157)
Supplement: S1 Text — (DOCX) [file pone.0180157.s006.docx]

## Determination of snail dry weight

The total wet weight of the snails was measured using live snails after removing excess water with a paper tissue. The wet weight was converted to dry weight using the following regression:

$y=0.5404x+0.6239$, R^2^=0.95, where y is the dry weight (mg) and x is the wet weight (mg). This regression was established using 40 snails with wet weight ranging from 7.4 to 92 mg; these snails were collected prior to the field experiment (year 1). The samples were dried at 60 °C for 72 h and weighed using a microbalance (±0.13 mg).
