# Supplementary material for: Metal contamination in harbours impacts life-history traits and metallothionein levels in snails
Source: PLoS One. 2017 Jul 3;12(7):e0180157. doi: 10.1371/journal.pone.0180157 (PMC5495383; doi:10.1371/journal.pone.0180157)
Supplement: S2 Text — (DOCX) [file pone.0180157.s007.docx]

## Quality control and calculation of metallothionein concentrations

The recovery of MT using commercial rabbit liver metallothionein MT-1 (Enzo) was 75 ± 7.8 %. The limit of detection (LOD) for MTs was calculated as 3 * standard deviation (of intercepts)/ average of slopes and was 0.03 nmol SH/mL.

The equation used for calculating MT from SH (1):

$conc(ug MT/g snail) = [conc SH (nmol/mL)/18] *MW* volume (mL)* dilution factor * [1/ snail weight (g)]$

where 18 is the number of cysteine residues and MW is the molecular weight of snail MT: 6620 g/mol (2).

## References

1. United Nations Environment Programme, editor. Manual on the biomarkers recommended for the Med Pol Biomonitoring Programme =: Manuel sur les biomarqueurs recommandes pour le programme de biosurveillance du Med Pol. Athens: United Nations Environment Programme; 1999. 92 p.

2. Dallinger R, Berger B, Hunziker PE, Birchler N, Hauer Cr, Käagi Jh. Purification and primary structure of snail metallothionein. Eur J Biochem. 1993;216(3):739–746.
