# Supplementary material for: Metal contamination in harbours impacts life-history traits and metallothionein levels in snails
Source: PLoS One. 2017 Jul 3;12(7):e0180157. doi: 10.1371/journal.pone.0180157 (PMC5495383; doi:10.1371/journal.pone.0180157)
Supplement: S3 Text — (DOCX) [file pone.0180157.s008.docx]

Between-group PCA

Analysis of inter-group variability was performed using the Between Group Principal Component Analyses (bgPCA), a method designed for exploring data with variance that can have distinct causes. In environmental studies, the method has been used to explore the relative influence of the sample location on the variability of the measures and the seasonal succession (1). In the context of our research questions, bgPCA was applied to provide an overview for the between-site variability in life history responses and measured environmental factors. It is fully plausible, however, that similar responses were related to different underlying environmental causes at different sites.

The analysis consists of a PCA run on the group-weighted average values of the variables using data matrices whose objects are gathered by groups. It seeks for axes of the centre of gravity space focusing on the between-group difference. In the ordination, the weight of a group equals the number of observations in this group. In our analysis, the groups were the sites. A bgPCA was computed for year 1 (Figure S6a) and year 2 (Figure S6b) separately using environmental (Cu, Zn, pH, salinity, TP and TN) and biological (growth, fecundity, mortality) variables.

The bgPCA plot showed a clear separation of the study sites based on the life histories and environmental variables (Figure S6). This distinction was evident in both years. In year 1, the first principal component (PC1) explained 65.2 % of the variance, with the strongest positive loadings for fecundity, RGR, pH and Cu_sed_ and the negative loadings for mortality and dissolved Cu. The second component (PC2) explained the remaining variance and was mostly associated with the positive loadings of dissolved Zn and TN and negative loadings of Zn_sed_ and salinity. In year 2, PC1 described 69.2 % of the variance, with the main positive loadings for mortality rate and dissolved Cu and the negative loadings for fecundity and RGR. PC2 explained 24.3 % of the variance and was mostly defined by nutrients, with negative loadings for TP and positive for TN. PC3 explained the remaining variance and was mostly associated with the positive loadings of metals in the sediment.

**Reference**

1. Tammert H, Tšertova N, Kiprovskaja J, Baty F, Nõges T, Kisand V. Contrasting seasonal and interannual environmental drivers in bacterial communities within a large shallow lake: evidence from a seven year survey. Aquat Microb Ecol. 2015 Apr 23;75(1):43–54.
